# Enhancing the Anaerobic Biodegradation of Petroleum Hydrocarbons in Soils with Electrically Conductive Materials

**DOI:** 10.3390/bioengineering10040441

**Published:** 2023-04-01

**Authors:** Carolina Cruz Viggi, Matteo Tucci, Marco Resitano, Valentina Palushi, Simona Crognale, Bruna Matturro, Marco Petrangeli Papini, Simona Rossetti, Federico Aulenta

**Affiliations:** 1 Water Research Institute (IRSA), National Research Council (CNR), 00010 Montelibretti, Italy; 2 National Biodiversity Future Center, 90133 Palermo, Italy; 3 Department of Chemistry, Sapienza University of Rome, 00185 Rome, Italy

**Keywords:** contaminated soil, electrically conductive materials, biochar, magnetite, petroleum hydrocarbons, bioremediation

## Abstract

Anaerobic bioremediation is a relevant process in the management of sites contaminated by petroleum hydrocarbons. Recently, interspecies electron transfer processes mediated by conductive minerals or particles have been proposed as mechanisms through which microbial species within a community share reducing equivalents to drive the syntrophic degradation of organic substrates, including hydrocarbons. Here, a microcosm study was set up to investigate the effect of different electrically conductive materials (ECMs) in enhancing the anaerobic biodegradation of hydrocarbons in historically contaminated soil. The results of a comprehensive suite of chemical and microbiological analyses evidenced that supplementing the soil with (5% *w*/*w*) magnetite nanoparticles or biochar particles is an effective strategy to accelerate the removal of selected hydrocarbons. In particular, in microcosms supplemented with ECMs, the removal of total petroleum hydrocarbons was enhanced by up to 50% relative to unamended controls. However, chemical analyses suggested that only a partial bioconversion of contaminants occurred and that longer treatment times would have probably been required to drive the biodegradation process to completion. On the other hand, biomolecular analyses confirmed the presence of several microorganisms and functional genes likely involved in hydrocarbon degradation. Furthermore, the selective enrichment of known electroactive bacteria (i.e., *Geobacter* and *Geothrix*) in microcosms amended with ECMs, clearly pointed to a possible role of DIET (Diet Interspecies Electron Transfer) processes in the observed removal of contaminants.

## 1. Introduction

Soil contamination from petroleum hydrocarbons (PH) is an extensive problem frequently linked to everyday industrial operations, such as oil exploration, extraction, processing, utilisation, disposal and accidental spills, although PH may naturally be present in subsurface environments [1,2].

Petroleum is composed of a highly complex combination of aromatic and aliphatic hydrocarbons, heterocycles, asphaltenes, and other (non-hydrocarbon) compounds. The non-polar fraction is mainly represented by saturated and aromatic hydrocarbons [3]. When an oil spill occurs, different physicochemical and/or biological strategies can be used to contrast the negative environmental impacts of PH. Physico-chemical methods are usually relatively expansive and can have an adverse effect on the residual soil quality and cause side pollution effects [2]. Bioremediation, on the other hand, takes advantage of the diverse metabolic abilities of autochthonous microorganisms, making it a more desirable option because of its minimal environmental impact, cost-effectiveness, versatility in treating different types of contamination, and potential for full mineralisation of the contaminants [3,4,5]. Anoxic conditions typically dominate in confined hydrocarbon-rich subsurface environments, such as soils, sediments, and aquifers. This is because the excess organic carbon present causes oxygen to be rapidly depleted, and the slow permeation from neighbouring toxic environments cannot keep up with the depletion rate [6,7]. In this context, anaerobic biodegradation of hydrocarbons has become an increasingly important process in the management of contaminated subsurface environments [8]. A large variety of aliphatic and aromatic hydrocarbons have been biodegraded under anaerobic conditions. Wartell and colleagues wrote an extensive review of the current knowledge about the biodegradation of PH under anaerobic conditions, where they presented a few dozen of examples of successful anaerobic hydrocarbon degradation studies [9]. In many anaerobic ecosystems, syntrophy assumes a primary role since it necessitates microbial species to function as a unified catalytic unit for the mutually beneficial metabolism of substrates [10]. As already mentioned, hydrocarbon-contaminated environments are usually anaerobic. Thus, methanogenic conditions are often established. Syntrophic processes involving the interspecies transfer of reducing equivalents (e.g., hydrogen, formate, or electrons) are typically required for the biodegradation of hydrocarbons to methane, and recent studies suggested that syntrophy trophic hydrocarbon metabolism can also occur under nitrate-, iron- or sulfate-reducing conditions [11,12,13]. In recent years, an alternative to the H_2_/formate transfer was found in the so-called direct interspecies electron transfer (DIET) [14,15], where bacteria exchange electrons directly via biological (e.g., multiheme cytochromes and/or pili) and non-biological electrical connections [16]. Non-biological electrically conductive materials (ECMs), including mineral particles and carbon materials, can enhance DIET in syntrophic cultures by serving as electron conduits [17].

Based on these considerations, DIET could be exploited to enhance the treatment of PH-contaminated soils. In fact, recent relevant publications evidenced the significance of DIET-driven hydrocarbon degradation in the field of biogeochemistry and bioremediation [18,19,20,21,22,23]. 

Here, a microcosm study was set up to evaluate the efficacy of different ECMs (i.e., magnetite nanoparticles and biochar) in enhancing the anaerobic biodegradation of PH in a historically contaminated soil sampled in a petrochemical site in central Italy and consisting of a complex mixture of aliphatic and aromatic hydrocarbons.

## 2. Materials and Methods

### 2.1. Soil Characteristics

Soil samples were obtained from a petrochemical site in Italy. Prior to use, the soil was sieved using a 2-mm screen and the hydrocarbon concentration was determined with the analytical procedure detailed below.

### 2.2. Electrically Conductive Materials

#### 2.2.1. Biochar

The biochar used in this study was prepared through the pyrolysis of pine wood carried out at approximately 850 °C [24]. The elemental composition of the obtained material was: 78% C; 4.18% Ca; 1.48% K; 0.67% Si; 0.64% Mn, and 0.46% Fe. The biochar was in the form of powder, and no further treatments were performed prior to its use in the experiments. Other biochar technical data were reported elsewhere [5,16,25].

#### 2.2.2. Magnetite Nanoparticles

The magnetite nanoparticles used in this study were synthesised as previously described [26]. In brief, 5.2 g of FeCl_3_ and 2.0 g of FeCl_2_ were dissolved into an acidic aqueous solution (HCl 0.4 M). Then the resulting solution was added drop-wise into an alkaline aqueous solution (NaOH 1.5 M) under vigorous mixing. A black precipitate of Fe_3_O_4_ (i.e., magnetite) was generated instantly at each drop addition. The precipitate was isolated using a magnetic field and purified by centrifugation. Finally, the particles were suspended in 0.25 L of deoxygenated water.

### 2.3. Experimental Setup

**Figure 1:**
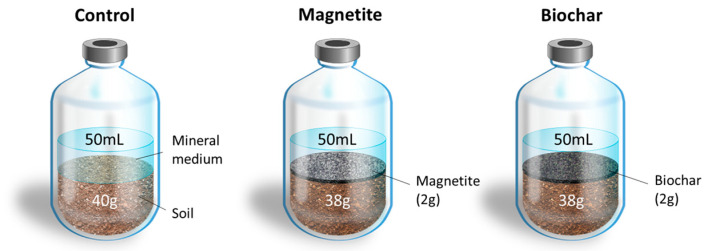
Sketch of the experimental setup.

The above-mentioned soil was used to set up sacrificial microcosms (24 in total) in 120 mL glass bottles (Figure 1). Each bottle was filled with the contaminated soil and anaerobic mineral medium, according to the amounts reported in Figure 1. The composition of the mineral medium used for the study is reported in the Supporting Information (Table S1). The medium had an electrical conductivity of 0.48 S/m, which falls within the range typically reported for contaminated aquifers [27].

Depending on the treatment, electrically conductive particles of biochar or magnetite (5% *w*/*w*) were added to the bottles. The amount of electrically conductive particles added to the microcosms (5% *w*/*w*) was comparable with previous studies reported in the literature [5,28,29,30,31,32]. After preparation, Teflon-coated butyl stoppers and aluminium seals were used to seal all the bottles, which were then purged with nitrogen (0.5 bar for 20 min) to create anaerobic conditions.

Three different treatments were set up, namely: the Control microcosms, containing the contaminated soil; the Magnetite microcosms, containing the contaminated soil supplemented with magnetite nanoparticles; the Biochar microcosms, containing the contaminated soil supplemented with biochar. 

Microcosms were statically incubated in the dark in a temperature-controlled room at 20 ± 1 °C. The gas composition of the headspace of each bottle was analysed weekly with a gas chromatograph equipped with a thermal conductivity detector (GC-TCD).

For each treatment, 8 identical bottles were prepared. At fixed times, one bottle from each treatment was sacrificed: aliquots (20 g) of soil were taken for hydrocarbon determination. The microbiome composition and key functional gene quantification were also determined on soil samples taken at the beginning and at the end of the experimentation.

### 2.4. Analytical Methods

#### 2.4.1. Gas Analyses

The concentration of hydrogen, methane and carbon dioxide was measured by injecting 50 µL of gaseous samples with a gastight syringe (Hamilton, Reno, NV, USA) into a gas-chromatograph equipped with a thermal conductivity detector (TCD, Agilent 8860, GC system, Santa Clara, CA, USA) as reported in the Supporting Information. Gas quantification was performed using external standards of hydrogen, methane and carbon dioxide. Standards were prepared to start from pure gases and suitably diluted to different concentrations with nitrogen.

#### 2.4.2. Hydrocarbons Determination

PH degradation was monitored at fixed time intervals by sacrificing one bottle from each treatment to collect approximately 20 g of soil. Hydrocarbons (i.e., PAH and TPH, Polycyclic Aromatic Hydrocarbons and Total Petroleum Hydrocarbons) were quantified by means of GC–MS upon microwave-assisted solid/liquid extraction. For each sample, duplicate extractions were performed in order to ensure reproducibility. 

To quantify TPH, the soil was air-dried at room temperature for 48 h on hexane-rinsed aluminium foil and then finely powdered using an agate mortar. Then, 5 g of dried samples were placed into 100 mL disposable glass vials, and 25 mL of a 1:1 acetone/hexane mixture was added to each vial. A Microwave Extraction system (Ethos X, Milestone, Bergamo, Italy) was used for the extractions (15 min at 110 °C and 6–10 bars). Extracts were cooled to room temperature and filtered through Florisil/Na_2_SO_4_ cartridges. Finally, extracts were concentrated to a final volume of 5 mL by evaporation under a gentle air stream. The extracts were analysed with a GC–MS (Perkin Elmer Clarus 680/600, Waltham, MA, USA) as reported in the Supporting Information. The TPH content was quantified by adding up the resolved and unresolved components that eluted from the GC capillary column between the retention times of *n*-C_10_ and *n*-C_40_. To do this, external calibration standards were prepared using solutions of diesel motor oil (Diesel #2/Motor oil, Restek, Centre, PA, USA) and diesel mineral oil (Diesel #2/Mineral oil, Restek, Centre, PA, USA) in hexane.

Concerning PAH determination, the methods for soil treatment and relative solvent extraction were the same as the ones reported for TPH. After being cooled to room temperature, the extracts were passed through Na_2_SO_4_ cartridges and concentrated to a final volume of 5 mL by evaporation under a gentle air stream. Then the extracts were analysed with a GC–MS (Perkin Elmer Clarus 680/600) according to the parameters reported in the Supporting Information. Quantification of PAH was performed using an external standard containing the 16 priority PAH Pollutants (PAH Calibration Mix, TraceCERT^®^, Merck, Rahway, NJ, USA).

### 2.5. DNA Extraction, Quantification of Key-Functional Genes and High-Throughput 16S rRNA Gene Sequencing

The contaminated soil used for the microcosms setup and soil samples were collected at the end of the experiments and immediately stored at −20 °C. A small amount of the collected soil (~0.5 g) was subjected to DNA extraction using the DNeasy PowerSoil Pro Kit (QIAGEN—Germantown, Maryland, USA) following the manufacturer’s instructions. The genomic DNA was used for the quantification of key-functional genes involved in the degradative pathways of petroleum hydrocarbons via Real-Time PCR (qPCR) following the procedures described in [5]. In particular, the genes involved in the anaerobic degradation of toluene (*bssA, bcrC, bzdN, bamB*) and alkanes (*assA*) were analysed [33,34,35,36]. Standard curves were created for the absolute quantification of each targeted gene, following the methods described previously. [37]. Data were analysed using CFX Manager™ Software 3.1 (Biorad, Milano, Italy), and the results were expressed as gene copies g^−1^ of soil. Averages and standard deviations were calculated on the three reactions of each sample. 

The genomic DNA was also used for the high-throughput sequencing of the V1-V3 region of the 16S rRNA gene of Bacteria (27F 5′-AGAGTTTGATCCTGGCTCAG-3′; 534R 5′-ATTACCGCGGCTGCTGG-3′) following the procedure for library preparation and sequencing described in [38]. The samples were paired-end sequenced (2 × 301 bp) on a MiSeq platform (Illumina) using a MiSeq Reagent kit v3, 600 cycles (Illumina, San Diego, CA, USA) following standard guidelines for sample preparation and loading. Phix control library was spiked at a concentration of 20%. Bioinformatics analysis was carried out using QIIME2 v. 2018.2 (accessed date 26 September 2022) [39] following the procedure described by Crognale et al. [40]. High-throughput sequencing of the V1-V3 region of the bacterial 16S rRNA gene yielded a total of 144,662 sequence reads after quality control and bioinformatic processing that resolved into 562 ASVs.

## 3. Results

### 3.1. Soil Characterization

**Figure 2:**
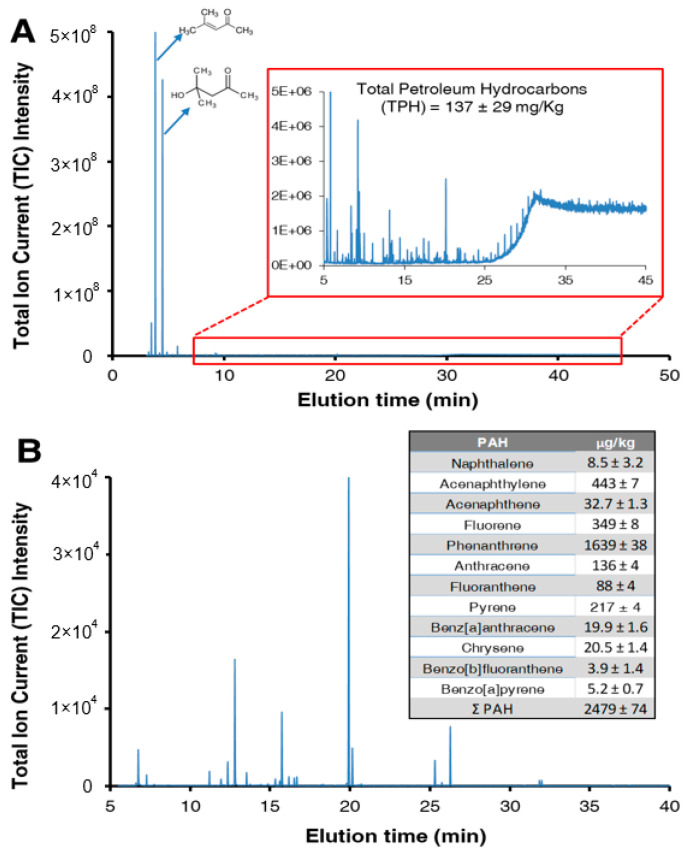
(**A**) Total Ion Current GC-MS chromatogram for the quantification of TPH in the contaminated soil. (**B**) Single Ion Monitoring GC-MS chromatogram for the quantification of polycyclic aromatic hydrocarbons (PAH) in the contaminated soil.

Figure 2A shows the Total Ion Current GC-MS chromatogram of the contaminated soil at the beginning of the experiments. The measured average TPH concentration (based on duplicate samples) was 137 ± 29 mg/kg. Interestingly, the chromatogram revealed the presence of two intense peaks, eluting from the column at 3.8 min and 4.4 min, whose mass spectrum matched with that of acetone isopropylidene (C_6_H_10_O, also known as mesityl oxide) and diacetone alcohol (C_6_H_12_O_2_), according to the National Institute of Standards and Technology (NIST) MS library. Although quantification of these ketones was not possible due to the lack of analytical standards, it is likely that these compounds were among the most abundant aliphatic hydrocarbons in the soil. However, it should be mentioned that these compounds were not accounted for in the TPH analysis since their retention time fell outside the integration window of TPH, which corresponded to the elution of *n*-C_10_ to *n*-C_40_ (i.e., from 7 min to 49 min). Furthermore, it is worth noting that both acetone isopropylidene and diacetone alcohol are solvents widely used in the chemical industry, especially for the production of varnishes, lacquers and paints, as well as for the production of lubricating oils (Haz-Map^®^, Information on Hazardous Chemicals and Occupational Diseases). Therefore, their presence in the soil is consistent with the past production activities of the petrochemical site from which the soil was sampled. Besides these compounds, the GC-MS chromatogram also revealed the presence of numerous linear and branched aliphatic hydrocarbons (though resulting in much less intense TIC signals) eluting from the column within 7–45 min (inset of Figure 2A).

Figure 2B shows the Single Ion Monitoring GC-MS chromatogram for the quantification of PAH in the contaminated soil at the beginning of the experiments. The measured total PAH concentration, based on analyses carried out on duplicate samples, was 2479 ± 74 µg/kg. Notably, the soil was found to contain 12 out of the 16 priority PAH (i.e., Benzo[*k*]fluoranthene, Dibenz[*a,h*]anthracene, Benzo[*ghi*]perylene and Indeno [1,2,3-c*d*]pyrene were not detected). Phenanthrene (1639 ± 38 µg/kg) was the most abundant PAH detected in the soil.

### 3.2. Effect of Electrically Conductive Particles on Hydrocarbons Degradation

At fixed times (i.e., on days 0, 26, 128 and 215), one bottle from each treatment was sacrificed to determine the remaining concentration of hydrocarbons in soils by GC–MS.

**Figure 3:**
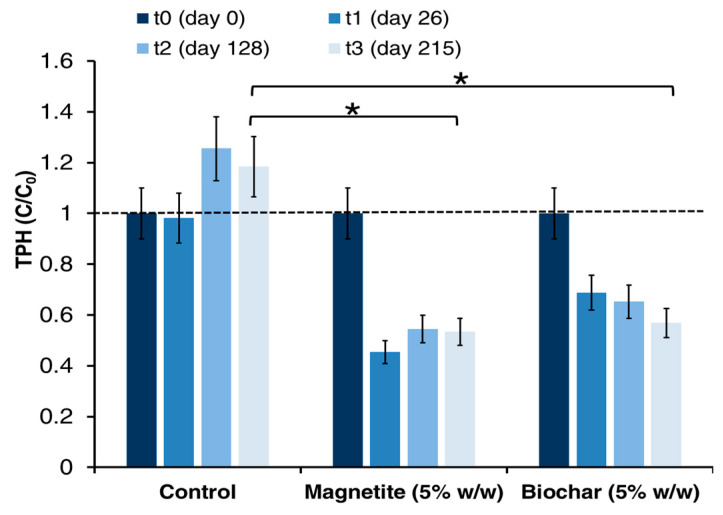
Total petroleum hydrocarbon (TPH) concentration over time in the different treatments. Error bars indicate the standard error of replicated samples. Significant differences were determined by two-tailed Student’s *t*-tests (* corresponds to *p* < 0.05).

As for TPH, microcosms not supplemented with ECMs (i.e., Control) displayed no apparent removal of contaminants over the whole incubation period (Figure 3). By contrast, a substantially higher (*p* < 0.05) TPH removal was observed in microcosms amended with magnetite nanoparticles (nearly 50% removal) and biochar (nearly 40% removal), respectively.

Notably, while almost no information is presently available on the impact of magnetite on anaerobic petroleum hydrocarbon biodegradation in soils [6], the stimulatory effect of biochar on TPH biodegradation is in line with earlier literature reports. In fact, previous studies suggested that adding biochar to soils contaminated with hydrocarbons can enhance both the extent and rate of TPH biodegradation [41,42,43,44]. In terms of removal rates, there were no apparent differences among treatments that were supplemented with magnetite and biochar, with values of about 0.45 mg/kg d^−1^, in either case.

**Figure 4:**
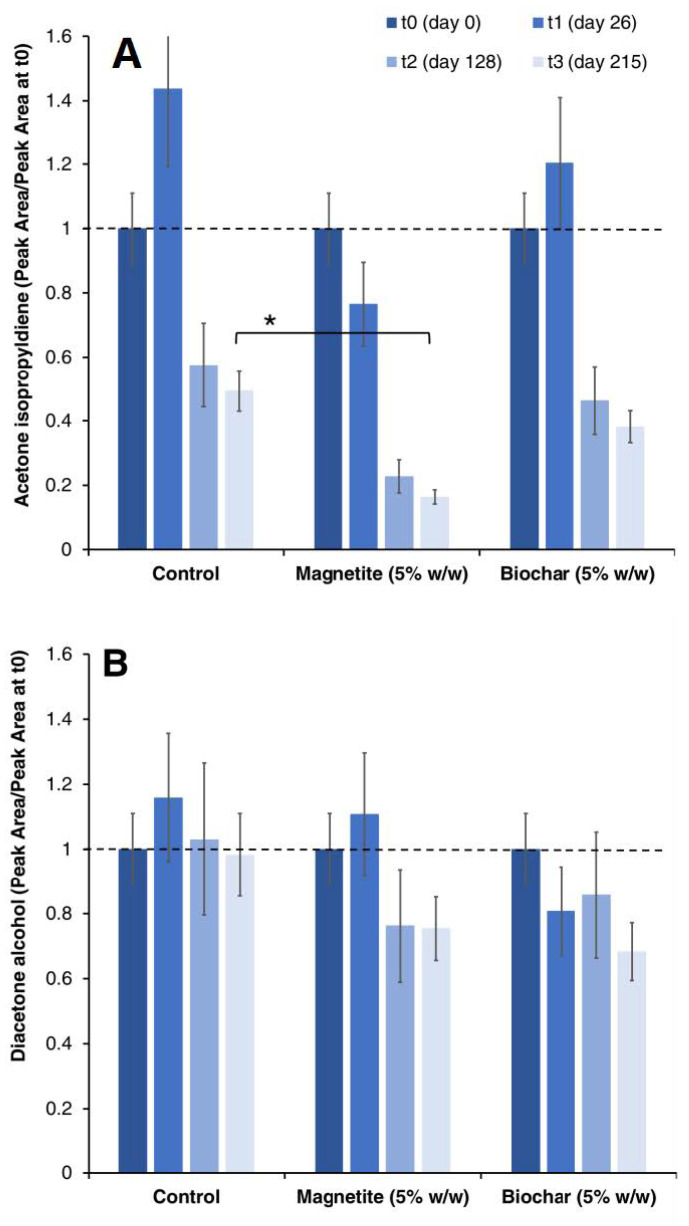
The relative concentration of acetone isopropylidene (**A**) and diacetone alcohol (**B**) over time in the different treatments. Error bars represent the standard error of replicated samples. Significant differences were determined by two-tailed Student’s *t*-tests (* corresponds to *p* < 0.05).

Figure 4 shows the change in relative concentrations of acetone isopropylidene and diacetone alcohol over time (calculated as the ratio of the peak areas) for the different treatments. After 215 days of incubation, substantial removal of acetone isopropylidene was observed in all treatments, although to a greater extent (nearly 85% removal) in the magnetite-supplemented microcosms (*p* < 0.05), compared to the unamended control (51%) and to the biochar-supplemented microcosms (62%). At the end of the incubation, the remaining concentrations of acetone isopropylidene in control and in the biochar-amended microcosms were not statistically different. Diacetone alcohol is a compound which may be formed from the acid-catalyzed reaction of acetone isopropylidene with water. It is not clear whether this compound was originally spilt or rather was formed directly in situ through biotic or abiotic reactions. Here, over the entire experimental period, the apparent removal of diacetone alcohol was rather limited (and statistically indistinguishable) in all treatments.

**Figure 5:**
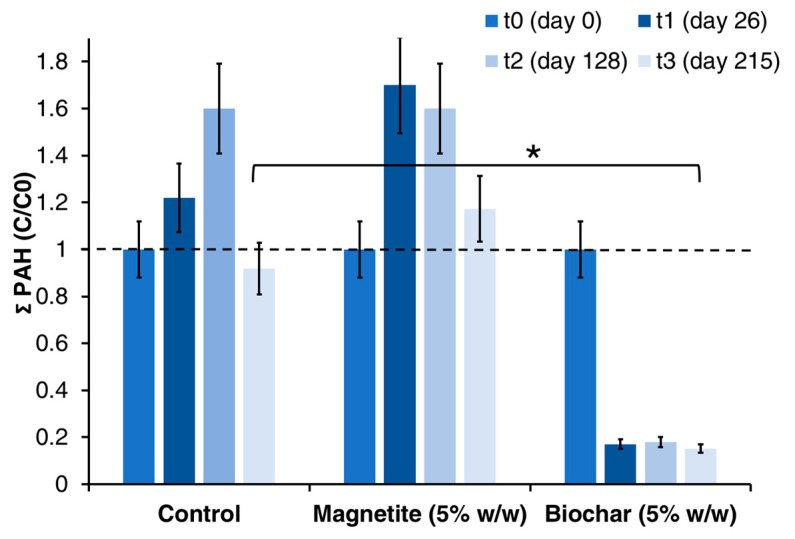
The relative concentration of total priority PAH detected in the soil over time in the different treatments. Error bars represent the SE of replicated samples. Significant differences were determined by two-tailed Student’s *t*-tests (* corresponds to *p* < 0.05).

Figure 5 shows the relative concentration of the priority PAH pollutants in the soil over time for the different treatments. The control and the magnetite-supplement microcosms displayed no apparent removal of contaminants throughout the whole experimental period, as well as important fluctuations in the relative concentration of PAH, which was apparently higher in correspondence to day 26 and day 128 (i.e., the 2nd and 3rd sampling times) with respect to the start of the experiment. These fluctuations were probably due to the not homogeneous distribution of contaminants within the soil. By contrast, a substantial removal (up to nearly 80%) was observed in the treatments supplemented with biochar.

Although PAH certainly has a high affinity for biochar, it is unlikely that adsorption was the exclusive removal mechanism for these contaminants. Indeed, the sample used for PAH analysis contained both the soil and the biochar. Therefore contaminants would have ultimately leached out from the soil and biochar upon solid-liquid extraction with the hexane-acetone mixture, carried out at a high temperature and in the presence of microwaves. Hence, it is more likely that the initial PAH adsorption over the biochar surface has favoured an effective co-localization of the contaminant and of the degrading microorganisms, thereby prompting a rapid and effective biodegradation process.

Biochar addition has been largely studied as a method to remediate soils contaminated by PAH by increasing their rate and extent of biodegradation [29,31,45]. In spite of that, the mechanisms by which biochar promotes PH biodegradation and contributes to the overall remediation process remain largely undefined [29]. Biochar can improve the water-holding capacity and fertility of soils due to its physicochemical properties [46]. Furthermore, biochar has been reported to regulate the microbial quality, activity, and composition of the microbial community by providing shelter and nutrients to soil microbes [47]. Finally, both biochar and magnetite have been studied as electron transfer catalysts in redox reactions of biogeochemical and environmental relevance [48,49] since they can facilitate syntrophic reactions by promoting Direct Interspecies Electron Transfer (DIET) processes [5,16,50].

### 3.3. Biogas Production

**Figure 6:**
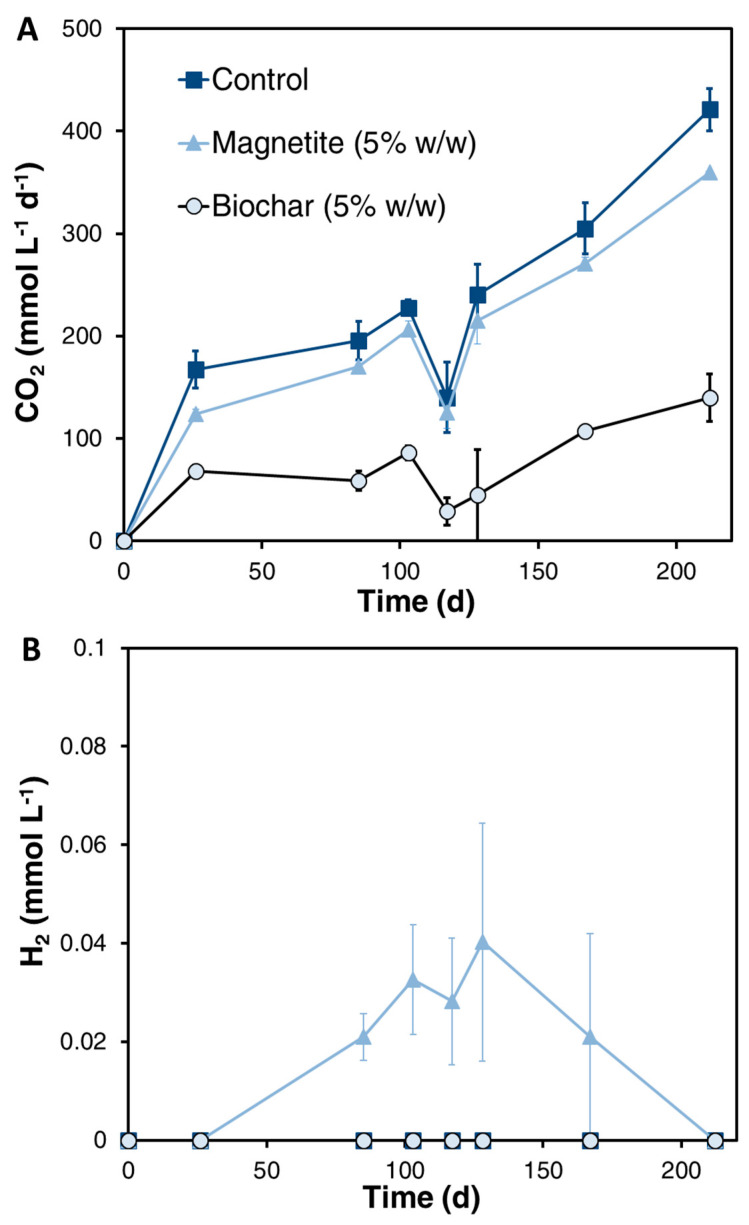
Time course of CO_2_ (**A**) and H_2_ (**B**) concentration in the headspace of the different microcosms during the whole experimental period. Error bars represent the SE of replicated samples.

Gas chromatographic analyses revealed a steady accumulation of carbon dioxide in the headspace of all microcosms, thus confirming the presence of an active metabolic activity of soil microorganisms. Apparently, a higher (but statistically indistinguishable) carbon dioxide production was noticed in control (420 mmolCO_2_ L^−1^ d^−1^) and in magnetite-supplemented microcosms (360 mmolCO_2_ L^−1^ d^−1^), whereas a lower production (140 mmol CO_2_ L^−1^ d^−1^) was observed in biochar-amended microcosms (Figure 6A). The fact that carbon dioxide production in control and in magnetite-supplemented microcosms was similar despite the different TPH removal suggests that TPH either contributed marginally to the overall CO_2_ production and/or was not completely mineralised. Similar considerations also apply to the biochar-supplemented microcosms: the lower apparent CO_2_ accumulation in the headspace with respect to the control microcosms might be due to the slight alkalinisation of the soil triggered by the biochar. Along this line, it is worth noting that recent studies have reported a decrease in soil respiration following biochar addition [51].

Methane, which is the other gaseous end-product of organic matter decomposition under anaerobic conditions, remained below the instrumental detection limit in all treatments during the whole experimental period. This finding provides a further indication that removed hydrocarbons were most likely only partially degraded.

Interestingly, a peak of hydrogen (Figure 6B) was observed in microcosms supplemented with magnetite nanoparticles (day 125), while it remained below the instrumental detection limit in control and in the biochar-supplemented microcosms. Since H_2_ is a key metabolic intermediate in the anaerobic degradation/fermentation of organic matter, it is likely that magnetite enhanced the fermentative conversion of the soil organic matter and anthropogenic contaminants, which is consistent with the measured hydrocarbon concentrations.

Overall, gas monitoring data provided important insights into the bioremediation process. In fact, they clearly highlighted that hydrocarbon removal was not associated with the complete mineralisation of contaminants to harmless end products, such as carbon dioxide and methane. On the contrary, the observed removal of contaminants was most likely incomplete and resulted in the accumulation of degradation intermediates whose further conversion to methane and carbon dioxide would have required longer incubation times.

### 3.4. Microbial Community Characterization

**Figure 7:**
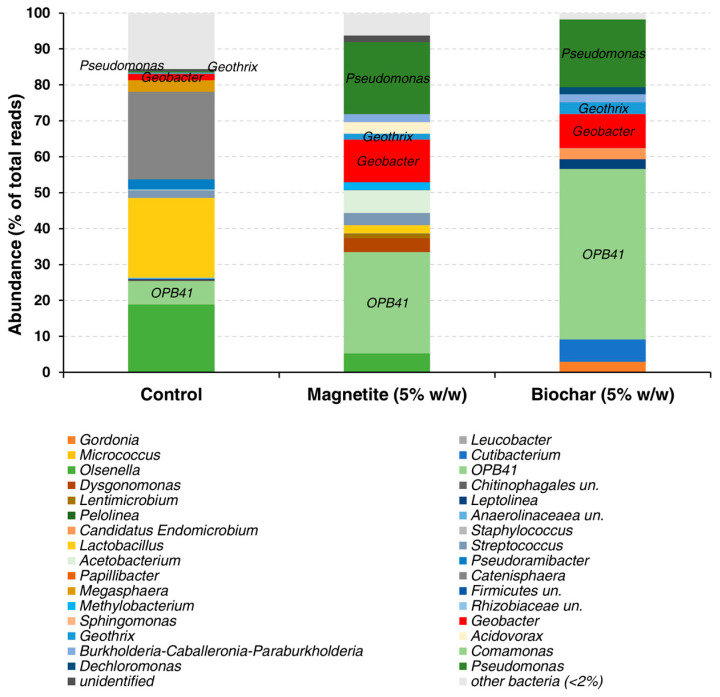
Bacterial community composition was revealed by the 16S rRNA gene amplicon sequencing. Data are expressed as relative abundance (% of total reads) of genera (≥2% in at least one sample) in the soil samples taken at the end of experiments.

The amplicon sequencing of the 16S rRNA gene (Figure 7) revealed important differences between the control microcosms and those supplemented with electrically conducted particles in terms of bacterial community composition. Remarkably, the presence of magnetite and biochar resulted in a substantial enrichment of well-known electroactive bacteria, such as *Geobacter* and *Geothrix* (collectively accounting for 1.9% in control vs. 13.5% and 12.6% in the magnetite- and biochar-supplemented microcosms, respectively), thus suggesting that conductive particles triggered DIET processes among community members. Besides that, microcosms supplemented with magnetite and biochar also resulted in a substantial enrichment of known hydrocarbons degraders, such as *Pseudomonas* (0.3% in the control vs. 20.1% and 18.8% in the magnetite- and biochar-supplemented microcosms, respectively), and *Burkholderiaceae* (nearly absent in the control while accounting up to 8.6% of total reads in magnetite and biochar supplemented microcosms) [52,53,54,55,56,57]. Finally, conductive particles resulted in a substantial enrichment of unidentified members of *Actinobacteria* order OPB41 (6.6% in the control vs. 28.3% and 47.5% in the magnetite- and biochar-supplemented microcosms, respectively), whose metabolic potentialities in TPH degradation have not been elucidated so far. Nevertheless, these microorganisms were previously reported in studies concerning the control hydrocarbon degradation [58,59]. Sequencing data have been deposited at DDBJ/ENA/GenBank under the BioProject PRJNA947826.

**Figure 8:**
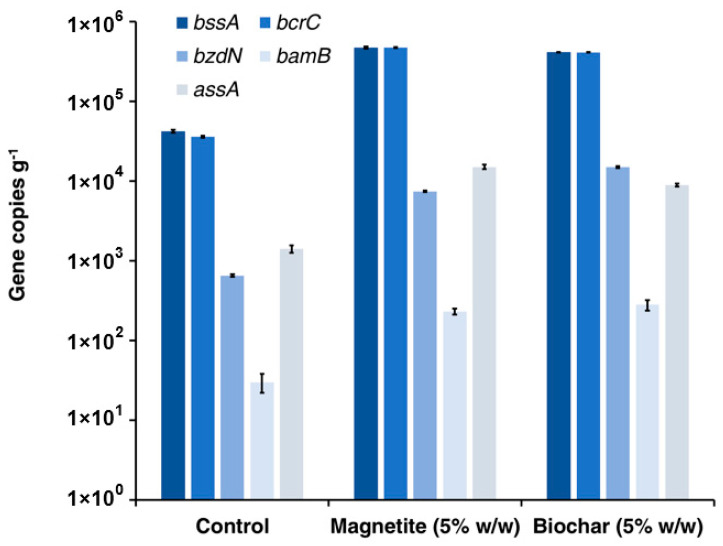
The abundance of key-functional genes involved in anaerobic toluene and alkanes degradation was estimated by qPCR in soil samples taken at the end of experiments. *bssA*, benzylsuccinate synthase; *bcrC* and *bzdN*, benzoyl CoA reductases class I; *bamB*, benzoyl CoA reductase class II; *assA*, alkylsuccinate/2-(1-methylalkyl) succinate synthase. Data are reported in the Log scale.

In order to gain a deeper understating of the actual bioremediation potential of the microbial communities occurring in the different microcosms, the analysis of selected functional genes was carried out. Specifically, the abundance of functional genes encoding for enzymes involved in the anaerobic degradation of the model aromatic hydrocarbon toluene (i.e., *bssA*, *bcrC*, *bzdN*, and *bamB*) and of alkanes (i.e., *assA*) was estimated by using qPCR assays. Even though all of these genes were detected in all microcosms, a higher abundance (>one order of magnitude) was observed in magnetite- and biochar-amended samples (Figure 8). In particular, the benzyl succinate synthase (*bssA*), a biomarker gene of anaerobic toluene degrading bacteria that use fumarate addition pathway [60], ranged between 4.2 × 10^4^ gene copies/g (control) and 5.1 × 10^5^ gene copies/g (magnetite-supplemented microcosms). A qualitatively similar trend was also observed for the genes encoding for the ATP-dependent class I benzoyl CoA reductases (*bcrC* and *bzdN)*, for the gene *bamB*, encoding for the ATP-independent class II benzoyl CoA reductases, as well as for the gene involved in *n*-alkanes degradation (*assA*).

Overall, the observed abundance of the key-functional genes involved in anaerobic hydrocarbon degradation pointed to the presence in the soils of a microbial community with a metabolic potential for petroleum hydrocarbon biodegradation, prone to be enhanced by the presence of conductive particles. This result is fully in agreement with the results from 16S rRNA gene amplicon sequencing.

## 4. Conclusions

This study demonstrated that supplementing soil historically contaminated by petroleum hydrocarbons with (5% *w*/*w*) electrically conductive magnetite or biochar particles is an effective strategy to accelerate the biodegradation of the pollutants present in the site. Despite the promising results in terms of contaminants removal, analytical measurements failed to identify the final anaerobic biodegradation product (e.g., methane). This suggests that only a partial bioconversion of contaminants occurred and that longer treatment times would have been required to drive the biodegradation process to completion. This hypothesis is supported by the amplicon sequencing of the 16S rRNA gene of the soil microbial communities, which confirmed the presence of several microorganisms and functional genes likely involved in PH degradation. Biomolecular analyses also revealed the selective enrichment of known electroactive bacteria (i.e., *Geobacter* and *Geothrix*) in microcosms amended with conductive particles, thus highlighting a possible role of DIET processes in the observed removal of contaminants. Clearly, further study will have to shed light on the mechanisms underlying the electrical interactions among microbes and particles, as well as on the pathways of contaminants’ transformation. This latter information is of utmost importance for the real-world application of the proposed bioremediation process. It is important to underline that, prior to moving to field testing, it will also be necessary to conduct ecotoxicity tests to provide a comprehensive assessment of the efficacy of the bioremediation process.

## Data Availability

The data presented in this study are available on request from the corresponding author.

## Supplementary Materials

The following supporting information can be downloaded at: https://www.mdpi.com/article/10.3390/bioengineering10040441/s1, Table S1: Composition of the mineral medium used for the study; Table S2: Set of primers used for qPCR; Paragraph 1: GC-TCD analyses of the gas phase; Paragraph 2: GC-MS parameters for the TPH analysis of the extracts; Paragraph 3: GC-MS parameters for the PAHs analysis of the extracts.
