# Supplementary material for: Enhancing the Anaerobic Biodegradation of Petroleum Hydrocarbons in Soils with Electrically Conductive Materials
Source: Bioengineering (Basel). 2023 Apr 1;10(4):441. doi: 10.3390/bioengineering10040441 (PMC10135592; doi:10.3390/bioengineering10040441)
Supplement: Supplementary file 1 [file bioengineering-10-00441-s001.zip › bioengineering-2285755-supplementary.pdf]

Enhancing the anaerobic biodegradation of petroleum hydrocarbons in soils with electrically conductive materials

Supporting information

Carolina Cruz Viggi[1,*], Matteo Tucci[1], Marco Resitano[1], Valentina Palushi[1], Simona Crognale[1,3], Bruna Matturro[1,3], Marco Petrangeli Papini[2], Simona Rossetti[1] and Federico Aulenta[1,3]

[1] Water Research Institute (IRSA), National Research Council (CNR), Via Salaria km 29300, 00015 Monterotondo (RM), Italy
[2] Department of Chemistry, Sapienza University of Rome, 00185 Rome, Italy
[3] National Biodiversity Future Center, Palermo 90133, Italy
* Correspondence: carolina.cruzviggi@irsa.cnr.it

Table S1. – Composition of the mineral medium used for the study.

Minerals	
**Compound**	**Concentration (g/L)**
NH_4Cl	0.5
$MgCl_2 \times 6H_2O$	0.1
$CaCl_2 \times 2H_2O$	0.05
K_2HPO_4	0.4
**Trace metals**	
**Compound**	**Concentration (mg/L)**
Nitrilotriacetic acid	4.5
$FeSO_4 \times 7H_2O$	0.556
$MnSO_4 \times H_2O$	0.086
$CoCl_2 \times 6H_2O$	0.17
$ZnSO_4 \times 7H_2O$	0.21
H_3BO_3	0.019
$NiCl_2$	0.02
Na_2MoO_4	0.01
**Vitamins**	
**Compound**	**Concentration (mg/L)**
Biotin (B7)	0.02
Folic acid (B9)	0.02
Pyridoxine (B6)	0.1
Thiamine (B1)	0.05
Riboflavin (B2)	0.05
Nicotinic acid (B3)	0.05
Pantothenic acid (B5)	0.05
Cyanocobalamin (B12)	0.002
4-aminobenzoic acid (B10)	0.05

Table S2. Set of primers used for qPCR.

Gene name	Primers ID	Primer sequences (5'-3')	Reference
**assA**	assA2F	YATGWACTGGCACGGMCA	[33]
	assA2R	GCRTTTTCMACCCAKGTA	
**bssA**	7772f	GACATGACCGACGCSATYCT	[36]
	8546r	TCGTCGTCRTTGCCCCAYTT	
**bcrC**	bcrCf	CGHATYCCRCGSTCGAC-CATCG	[34]
	bcrCr	CGGATCGGCTGCATCTGGCC	
**bzdN**	bzdNf	GAGCCGCACATCTTCGGCAT	[34]
	bzdNr	TRTGVRCCG-GRTARTCCTTSGTCGG	
**bamB**	bamBf	ATGMGGTAYGSAGARACHGG	[35]
	bamBr	CCSGCRWRYTTCADYTCCG	

1. GC-TCD analyses of the gas phase

50 μL of gaseous samples were injected in a gas-chromatograph equipped with a thermal conductivity detector (TCD, Agilent 8860, GC system USA); column: Agilent Carboxen 1000 stainless steel packed (3.05 m × 0.32 cm, OD, 2 mm ID, Carboxen-1000 packing, mesh size 60/80, pre-conditioned); carrier gas: Nitrogen 10 mL/min; Injection Temp: 200 °C; Interface Temp: 300 °C; Oven Temp Program: 100°C for 10.0 min.

2. GC-MS parameters for the TPH analysis of the extracts

The extract (1 μL) was injected (in pulsed split-less mode) into a GC–MS (Perkin Elmer Clarus 680/600; column: HP-5 MS (Agilent) 30 m, ID 0.25 mm, 0.25 mm film thickness; carrier gas: helium 1 mL/min; Inj T: 310 °C; Interface T: 280 °C; Oven T Program: 50 °C for 0.0 min, then 20 °C/min to 100 °C, then 5 °C/min to 300 °C, then 300 °C for 2.5 min. MS method: Full Scan mode, mass range 35–650 m/z)

3. GC-MS parameters for the PAHs analysis of the extracts

The extract (1 μL) was injected (in pulsed split-less mode) into a GC–MS (Perkin Elmer Clarus 680/600; column: HP-5 MS (Agilent) 30 m, ID 0.25 mm, 0.25 mm film thickness; carrier gas: helium 1 mL/min; Inj T: 310 °C; Interface T: 280 °C; Oven T Program: 50 °C for 0.0 min, then 20 °C/min to 100 °C, then 5 °C/min to 300 °C, then 300 °C for 2.5 min. MS method: Selected Ion Monitoring (m/z: 78, 91, 128, 152, 154, 166, 178, 202, 228, 252, 276, 278).

REFERENCES

33. Aitken, C.; Jones, D.; Maguire, M.; Gray, N.; Sherry, A.; Bowler, B.; Ditchfield, A.; Larter, S.; Head, I. Evidence that crude oil alkane activation proceeds by different mechanisms under sulfate-reducing and methanogenic conditions. *Geochim. Cosmochim. Acta* **2013**, *109*, 162–174. https://doi.org/10.1016/j.gca.2013.01.031.
34. Kuntze, K.; Vogt, C.; Richnow, H.-H.; Boll, M. Combined Application of PCR-Based Functional Assays for the Detection of Aromatic-Compound-Degrading Anaerobes. *Appl. Environ. Microbiol.* **2011**, *77*, 5056–5061. https://doi.org/10.1128/aem.00335-11.
35. Löffler, C.; Kuntze, K.; Vazquez, J.R.; Rugor, A.; Kung, J.W.; Böttcher, A.; Boll, M. Occurrence, genes and expression of the W/Se-containing class II benzoyl-coenzyme A reductases in anaerobic bacteria. *Environ. Microbiol.* **2010**, *13*, 696–709. https://doi.org/10.1111/j.1462-2920.2010.02374.x.
36. Winderl, C.; Schaefer, S.; Lueders, T. Detection of anaerobic toluene and hydrocarbon degraders in contaminated aquifers using benzylsuccinate synthase (bssA) genes as a functional marker. *Environ. Microbiol.* **2007**, *9*, 1035–1046. https://doi.org/10.1111/j.1462-2920.2006.01230.x.
